# Sorafenib Alleviates Inflammatory Signaling of Tumor Microenvironment in Precancerous Lung Injuries

**DOI:** 10.3390/ph16020221

**Published:** 2023-02-01

**Authors:** Betul Cicek, Ahmet Hacimuftuoglu, Mehmet Kuzucu, Ahmet Cetin, Yesim Yeni, Sidika Genc, Serkan Yildirim, Ismail Bolat, Mecit Kantarci, Mustafa Gul, Serhat Hayme, Dimitris Matthaios, Dimitra P. Vageli, Sotirios G. Doukas, Aristidis Tsatsakis, Ali Taghizadehghalehjoughi

**Affiliations:** 1 Faculty of Medicine, Department of Physiology, Erzincan Binali Yildirim University, Erzincan 24100, Turkey; 2 Faculty of Medicine, Department of Medical Pharmacology, Ataturk University, Erzurum 25240, Turkey; 3 Faculty of Arts and Sciences, Department of Biology, Erzincan Binali Yildirim University, Erzincan 24100, Turkey; 4 Department of Biology, Graduate School of Natural and Applied Sciences, Erzincan Binali Yildirim University, 24100 Erzincan, Turkey; 5 Faculty of Medicine, Department of Medical Pharmacology, Malatya Turgut Ozal University, Malatya 44210, Turkey; 6 Faculty of Medicine, Department of Medical Pharmacology, Bilecik Seyh Edebali University, Bilecik 11230, Turkey; 7 Faculty of Veterinary, Department of Pathology, Ataturk University, Erzurum 25240, Turkey; 8 Faculty of Medicine, Department of Radiology, Erzincan Binali Yildirim University, Erzincan 24100, Turkey; 9 Faculty of Medicine, Department of Radiology, Ataturk University, Erzurum 25240, Turkey; 10 Faculty of Medicine, Department of Physiology, Ataturk University, Erzurum 25240, Turkey; 11 Faculty of Medicine, Department of Biostatistics, Erzincan Binali Yildirim University, Erzincan 24100, Turkey; 12 Oncology Department, General Hospital of Rhodos, 85100 Rhodos, Greece; 13 Yale Larynx Laboratory, Department of Surgery (Otololaryngology), Yale School of Medicine, Yale University, New Havan, CT 06510, USA; 14 Department of Internal Medicine, Division of Gastroenterology, Rutgers/Saint Peter’s University Hospital, New Brunswick, NJ 08901, USA; 15 Department of Forensic Sciences and Toxicology, Faculty of Medicine, University of Crete, 71003 Heraklion, Greece

**Keywords:** lung carcinogenesis, diethylnitrosamine, inflammation, SOX-2, COX-2, JNK

## Abstract

According to population-based studies, lung cancer is the prominent reason for cancer-related mortality worldwide in males and is also rising in females at an alarming rate. Sorafenib (SOR), which is approved for the treatment of hepatocellular carcinoma and renal cell carcinoma, is a multitargeted protein kinase inhibitor. Additionally, SOR is the subject of interest for preclinical and clinical trials in lung cancer. This study was designed to assess in vivo the possible effects of sorafenib (SOR) in diethylnitrosamine (DEN)-induced lung carcinogenesis and examine its probable mechanisms of action. A total of 30 adult male rats were divided into three groups (1) control, (2) DEN, and (3) DEN + SOR. The chemical induction of lung carcinogenesis was performed by injection of DEN intraperitoneally at 150 mg/kg once a week for two weeks. The DEN-administered rats were co-treated with SOR of 10 mg/kg by oral gavage for 42 alternate days. Serum and lung tissue samples were analyzed to determine SRY-box transcription factor 2 (SOX-2) levels. The tumor necrosis factor alpha (TNF-α) and interleukin-1 beta (IL-1β) levels were measured in lung tissue supernatants. Lung sections were analyzed for cyclooxygenase-2 (COX-2) and c-Jun N-terminal kinase (JNK) histopathologically. In addition, cyclooxygenase-2 (COX-2) and c-Jun N-terminal kinase (JNK) were analyzed by immunohistochemistry and immunofluorescence methods, respectively. SOR reduced the level of SOX-2 that maintenance of cancer stemness and tumorigenicity, and TNF-α and IL-1β levels. Histopathological analysis demonstrated widespread inflammatory cell infiltration, disorganized alveolar structure, hyperemia in the vessels, and thickened alveolar walls in DEN-induced rats. The damage was markedly reduced upon SOR treatment. Further, immunohistochemical and immunofluorescence analysis also revealed increased expression of COX-2 and JNK expression in DEN-intoxicated rats. However, SOR treatment alleviated the expression of these inflammatory markers in DEN-induced lung carcinogenesis. These findings suggested that SOR inhibits DEN-induced lung precancerous lesions through decreased inflammation with concomitant in reduced SOX-2 levels, which enables the maintenance of cancer stem cell properties.

## 1. Introduction

Nitrosamines are assessed as a considerable class of environmental carcinogens. Human is subject to nitrosamines from agricultural chemicals, tobacco products, pharmaceutical preparations, cosmetics, and food preservatives [1,2]. Additionally, these toxic chemicals are used to induce malignant tumors in experimental animals [1]. Among the various potent environmental carcinogens, diethylnitrosamine (DEN) is widely used in in vivo cancer models including the esophagus, liver, and lungs of its genotoxic, carcinogenic, and mutagenic potential [3,4,5]. The bioactivation of DEN by CYP450 enzymes leads to DEN transforming into a strong alkylating agent. This form of DEN adducts in the DNA, which causes the generation of ROS in turn to cause oxidative stress-mediated cytotoxicity, mutagenicity, and carcinogenicity [6,7]. In addition, it is reported that DEN enhances the cell proliferation cycle with pulmonary necrosis, increased lipid peroxidation, depletion of endogenous antioxidants, induce free radical production, cell damage, and lung tumor development [7].

Lung cancer remains the largest cause of cancer-related deaths worldwide with about 2.21 million new cases each year [8]. Chemotherapy is the standard therapeutic approach for most lung cancer patients; however, it does not effectively improve life expectancy due to drug resistance [9,10]. Although anticancer agents attack the tumor by inducing apoptotic cell death, the cancer cells can develop a resistance to apoptosis, decreasing their effectiveness [11,12]. Despite billions of dollars invested in cancer research, the death rates remained significantly high for many types of cancer. For that reason, the relationship between the mechanism of carcinogenesis, cancer origins, and targeted therapies has been questioned [9,12,13].

Considering oncogenic signaling pathways, the relationship between inflammatory response and cancer development is particularly noteworthy [14,15]. The common factor among numerous carcinogenic agents is the activation of genes that control inflammatory cell signaling pathways and these signals control all aspects of the cancer process [15,16]. Of these pathways, the role of c-Jun N-terminal kinase (JNK) signaling is considered to be a potentially suitable target for the treatment of inflammatory conditions [16,17,18] On the other hand, JNK signaling has two faces in cancer. JNK is associated with cancer cell apoptosis; however, emerging evidence demonstrated that the JNK pathway promotes cancer cell survival [19,20]. Recent data have provided direct evidence that the mucosa of the upper aerodigestive tract under chronic exposure to tobacco smoke components with or without the combination of gastroduodenal refluxate can induce premalignant lesions and deregulated DNA repair mechanisms, leading to invasive cancer through the activation of cancer-related inflammatory molecules, such as NF-kB and related oncogenic pathways [21,22,23]. In addition, previous preclinical and clinical findings from aggressive human upper aerodigestive tract cancer showed that cancer-related pre-inflammatory genes, such as TNF-α, and IL-1β, were significantly overexpressed during this process [24,25,26]. Another inflammatory parameter is the inducible cyclooxygenase (COX)-2 enzyme that catalyzes prostaglandin synthesis, which is the main mediator of inflammation and angiogenesis [19,27]. Overexpression of COX-2 has been detected in lung cancer and found to relate to tumor growth rate, as well as the resistance of cancer cells to conventional chemotherapy [28,29,30].

The second critically important condition responsible for the cancer process is the presence of cancer stem cells (CSCs) [31]. CSCs are a small population of cells in tumors that have self-renewal, infinite proliferation capability, and lead to resistance to conventional anticancer agents [31,32]. SOX-2, also known as sex-determining region Y (SRY)-box-2, is a transcription factor that is involved in the maintenance of CSC characteristics [33]. SOX-2 positively affects stem-like cells responsible for the initiation, maintenance, metastasis, and relapse of lung tumors [33,34]. In addition, inflammatory markers such as COX-2 are co-expressed with CSC markers including SOX-2 in cancer [33,35]. In genomic alterations in lung cancer, SOX-2 is considered a potential target for therapeutic intervention [30,36].

Inflammation is essential not only for cancer induction through its mutagenic effects on stem cell DNA but also because the subsequent progression of tumors is largely determined by the tumor microenvironment [15]. Exposure of stem cells’ DNA to or prolonged, unrepaired assaults by proinflammatory cytokines and chemokines can lead to genetic mutations that over time can convert a somatic stem cell into a cancer stem cell [16]. Although chemotherapy may slow and/or inhibit tumor growth, it may have little effect on overall patient survival [9]. Therefore, directing the therapy to inflammation and cancer stem cells may significantly enhance the treatment’s effectiveness. Recent data suggest the effectiveness of targeted therapies in various types of cancer such as breast and liver [37,38].

Sorafenib (NEXAVAR; SOR) is a multi-target protein kinase inhibitor approved for the treatment of hepatocellular carcinoma and advanced renal cell carcinoma [39,40]. Although SOR has not been approved for lung cancer, it is still the subject of extensive research related to lung cancer [41,42,43]. Results of the retrospective analysis reported that SOR significantly prolongs the recurrence-free survival of cancer patients. The efficacy of SOR is quite high even in patients who failed other treatments o or had only minimal response to chemotherapy [44]. It is a multitargeted molecule demonstrating its action thanks to the inhibition of proliferation and angiogenesis of tumor cells by its multi-kinase inhibitory property [45]. It also demonstrated that SOR in combination with antitumor mesenchymal stem cells potently inhibited tumor growth and preserved the antitumor-associated anti-inflammatory effects of mesenchymal stem cells [46]. Although previous studies suggested the efficacy of SOR against lung cancer [41,43] to the best of our knowledge, there is no study evaluating the relationship between JNK and COX-2 signaling and SOX-2 in the lung premalignant microenvironment. Therefore, we aimed to determine the mechanistic effect of SOR in DEN-induced lung carcinogenesis using a rat model. 

## 2. Results

### 2.1. Effects of SOR on Lung and Serum SOX-2 Levels in DEN-Induced Lung Carcinogenesis in Rats

**Figure 1:**
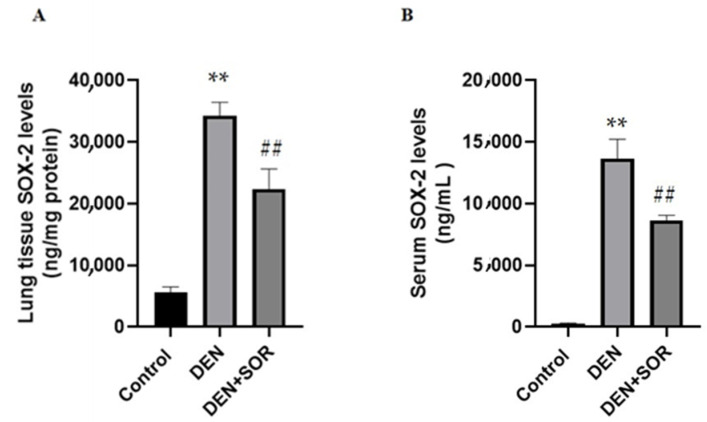
Effects of SOR on lung tissue (**A**) and serum (**B**) SOX-2 levels. (*n* = 10) Data are expressed as the mean ± standard deviation (mean ± 2 SD) ** *p* < 0.001 compared with the control group, ## *p* < 0.001 compared with DEN group DEN: diethylnitrosamine; SOR: Sorafenib; SOX-2: Sex-determining region Y (SRY)-box 2.

Compared to the controls (5.55 ± 0.94 ng/mg protein), in lung tissue SOX-2 protein levels were significantly increased in the DEN group (34.29 ± 2.14 ng/mg protein, *p* < 0.001). The DEN-induced elevation in SOX-2 levels in lung tissue was restored markedly in rats that received SOR treatment (22.36 ± 3.27 ng/mg protein, *p* < 0.001), (Figure 1A).

In control rats, the serum SOX-2 level was 0.25 ± 0.07 ng/mL which was increased significantly in DEN-injected rats (13.64 ± 1.59 ng/mL, *p* < 0.001). The DEN-induced increases in serum SOX-2 level were reduced (8.64 ± 0.41 ng/mL, *p* < 0.001) treatments of SOR (Figure 1B).

### 2.2. Effect of SOR Treatment on Lung TNF-α Levels in DEN-Induced Lung Carcinogenesis in Rats

**Figure 2:**
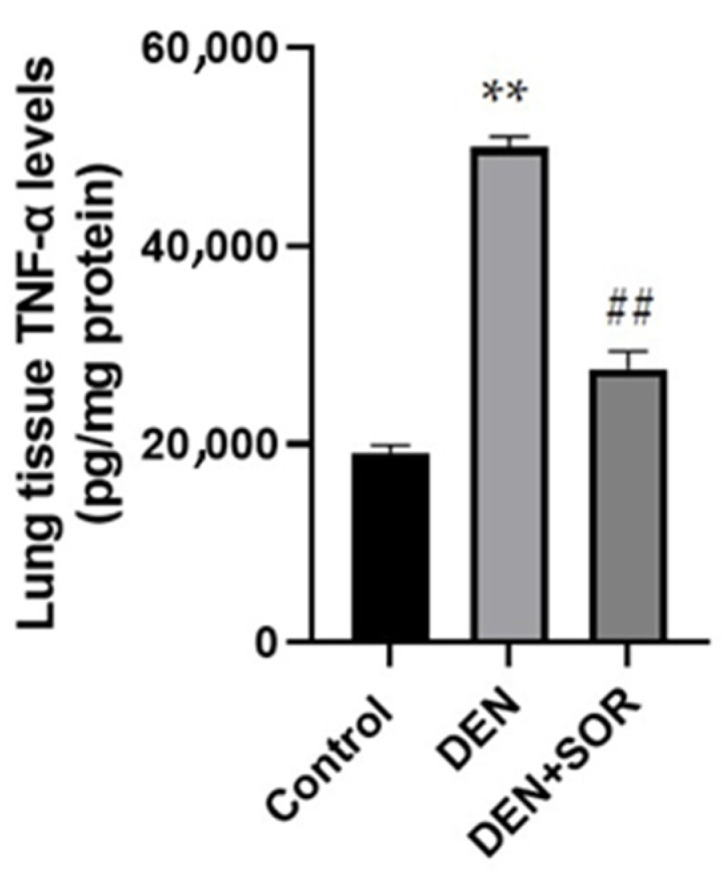
Effects of SOR on TNF-α in lung tissue. (*n* = 10) Data are expressed as mean ± standard deviation (mean ± 2 SD). ** *p*  <  0.001 compared with control group, ## *p*  <  0.001 compared with DEN group DEN: diethylnitrosamine; SOR: Sorafenib; TNF-α: Tumor necrosis factor alpha.

Compared with the control animals (19.09 ± 0.84 pg/mg protein), DEN elevated the TNF-α protein level in lung tissue significantly (50.06 ± 1.00 pg/mg protein, *p* < 0.001). The DEN-induced elevation of TNF-α protein level was prevented by SOR treatment (27.64 ± 1.75 pg/mg protein, *p* < 0.001), (Figure 2).

### 2.3. Effect of SOR Treatment on Lung IL-1β Levels in DEN-Induced Lung Carcinogenesis in Rats

**Figure 3:**
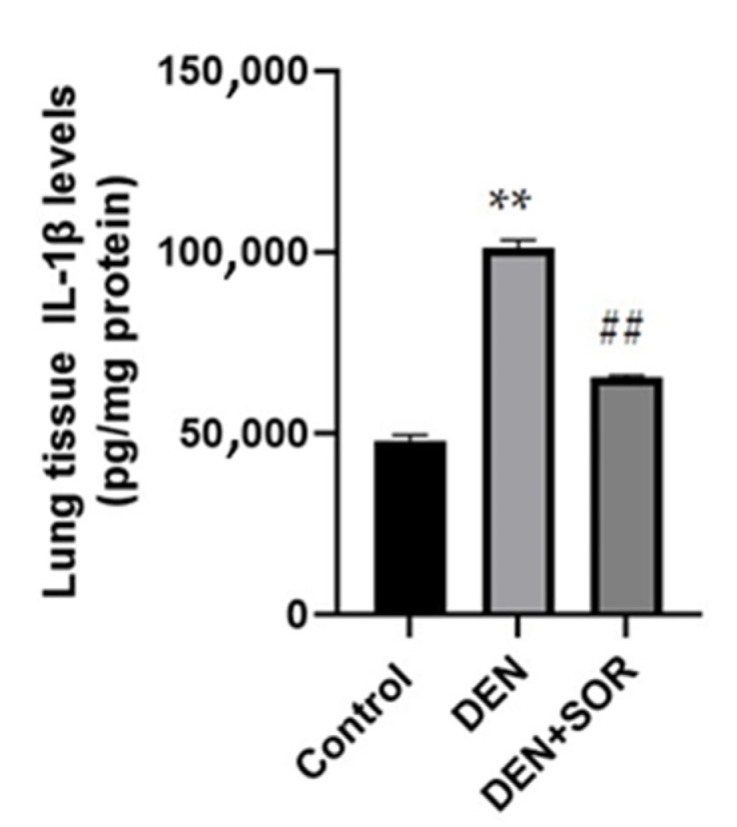
Effects of SOR on IL-1β in lung tissue. (*n* = 10) Data are expressed as mean ±  standard deviation (mean ± 2 SD). ** *p* < 0.001 compared with the control group, ## *p* < 0.001 compared with DEN group DEN: diethylnitrosamine; SOR: Sorafenib; IL-1β: Interleukin-1 beta.

In control rats, the lung tissue IL-1β level was 47.85 ± 1.65 pg/mg protein which was elevated marked in DEN-injected rats (100.95 ± 2.35 mg protein, *p* < 0.001). The DEN-induced rises in lung tissue IL-1β levels were reduced (65.26 ± 0.85 pg/mg protein, *p* < 0.001) with treatments of SOR (Figure 3).

### 2.4. Histopathological Examination of Lung

**Figure 4:**
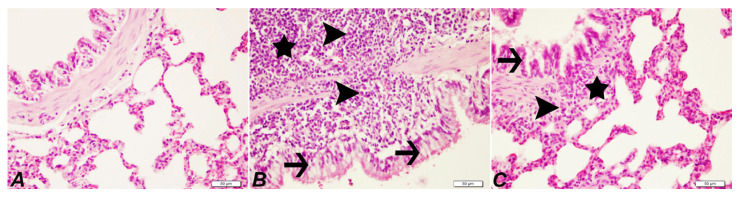
Lung histopathology of experimental groups (hematoxylin and eosin staining (40×), Bar: 50 µm. Control group (**A**), normal histological appearance; DEN group (**B**), peribronchiolar lymphohistiocytic cell infiltration (star), alveolar macrophages (arrowhead), and differentiation disorders due to epithelial degeneration (arrow); peribronchiolar lymphohistiocytic cell infiltration; DEN + SOR group (**C**), peribronchiolar cell infiltrations (star), alveolar macrophages (arrowhead) and epithelial degeneration (arrow). Control: healthy group (no treatment), DEN: diethylnitrosamine group, DEN + SOR: DEN + 10 mg/kg sorafenib.

Control group: The lung tissue of healthy rats was observed to have a normal histological structure. DEN group: Severe peribronchiolar cell infiltrations, severe exudation, and alveolar macrophages in the alveolar lumens, proliferation and degeneration in the bronchiole epithelium, severe thickening due to cell proliferation in the interstitial spaces, and severe hyperemia in the vessels were observed. DEN + SOR group: Compared with the DEN group, the treatment of SOR had partial signs of improvement. Mild peribronchiolar cell infiltration, mild thickening of the interstitial spaces, and moderate hyperemia in the vessels were detected (Figure 4 and Table 1).

### 2.5. Effects of SOR on the COX-2 Levels in Lung Tissue

**Figure 5:**
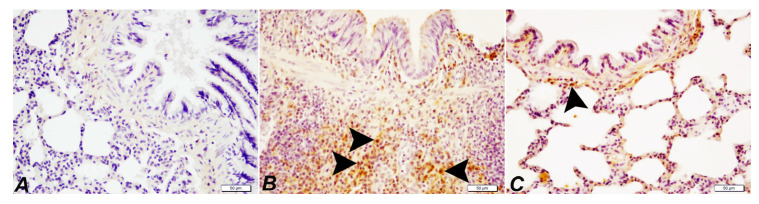
Immunohistochemical staining of COX-2 in experimental groups in lung tissue (×40), Bar: 50 µm. Control group (**A**), negative COX-2 expression; DEN group (**B**), Intracytoplasmic COX-2 expressions in lymphocytes and macrophages due to severe inflammatory reactions in the lung tissue (arrowhead); DEN + SOR group (**C**), COX-2 expression (arrowhead). Control: healthy group (no treatment), DEN: diethylnitrosamine group, DEN + SOR: DEN + 10 mg/kg sorafenib.

Control group: The lung tissue of healthy rats was observed immunohistochemically and found negative COX-2 expression. DEN group: As a result of DEN application, a severe level of COX-2 expression in peribronchiolar cells, alveolar lumens, and perivascular areas was determined. DEN + SOR group: When SOR treatment was applied to DEN groups, a marked decline in COX-2 expression was detected in peribronchiolar cells, around bronchial bronchioles and alveoli (Figure 5 and Table 2).

### 2.6. Effects of SOR on the JNK Levels in Lung Tissue

**Figure 6:**
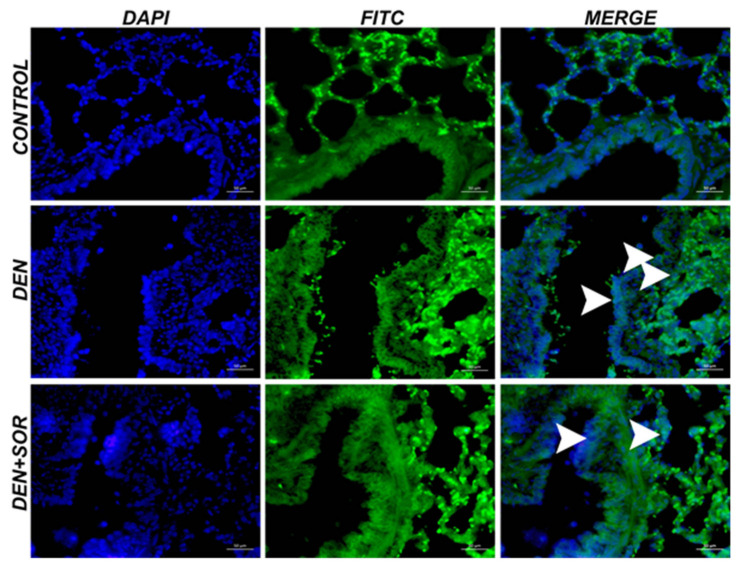
Immunofluorescence staining of JNK in experimental groups in lung tissue (×40), Bar: 50 µm. Control group, negative JNK expression; DEN group, Intracytoplasmic expression of JNK in epithelial cells due to the effect of the carcinogen DEN on the lungs (arrowhead); DEN + SOR group, JNK expression (arrowhead). Control: healthy group (no treatment), DEN: diethylnitrosamine group, DEN + SOR: DEN + 10 mg/kg sorafenib.

Control group: the lung tissue of healthy rats was examined by immunofluorescence and negative JNK expression was observed. DEN group: as a result of the DEN application, severe JNK expression was observed in the bronchi, bronchioles, and alveolar epithelium. DEN + SOR group: when SOR treatment was applied to DEN groups, a significant reduction in JNK expression was present in bronchi, bronchioles, and alveolar epithelium (Figure 6 and Table 3).

## 3. Discussion

In order to control the emergence and progression of cancer, it is necessary to study the survival mechanisms in cancer stem cells and the role of the tumor’s inflammatory microenvironment [15,16,30]. In contrast to traditional cytotoxic chemotherapies, targeted cancer therapies seem to have higher sensitivity and a more favorable side effect profile [47]. In this study, we analyzed the effects of SOR treatment on DEN-induced lung carcinogenesis in rats. The current study reported that SOR significantly inhibits DEN-induced lung early oncogenic events by decreasing the inflammatory parameters in the precancerous microenvironment and the level of SOX-2 protein, which mediates the self-renewal of cancer stem cells.

DEN is a well-known and commonly used chemical compound carcinogen for the in vivo induction of malignancies in the esophagus, liver, and lungs [3,4,5]. Some literature data demonstrated that DEN may be a potent lung carcinogen in strains with a higher incidence of lung tumors than other cancers [5,7,48]. We selected a dose of 150 mg/kg DEN based on previous in vivo studies [7,49], which demonstrated that the application of DEN at this dose remarkably reflects the pathological and biochemical findings of lung cancer in humans. Sivalingam et al. demonstrated that the DEN application in rats results in disorganized alveolar structure, interalveolar inflammatory cells, thickened alveolar walls, and red blood cells distributed in alveolar cavities [7]. In parallel with the previous literature, we determined that DEN administration significantly increased histopathological scores and inflammation in the present study. Lung tissues displayed extensive pathological changes in the DEN group. Widespread inflammatory cell infiltration, thickened alveolar wall, proliferation, degeneration in the bronchiole epithelium, and hyperemia in the vessels were observed in the DEN group. In contrast to the DEN group, the histopathological scores decreased and partial histopathological improvement was observed in the group treated with SOR.

Chronic inflammation is often a hallmark of tumor initiation and progression. JNK plays an important role in these inflammation-related events [15]. Many studies demonstrated that JNK is a positive regulator of tumor growth in the lung tissue of experimental models [50,51]. In line with such roles of JNK, it has been documented that JNK maintains the tumor-initiating capacity of lung stem cells and another role has been added to it as “one of the pre-tumor roles of JNK” [51]. Apart from this, JNK has activated the inflammatory lung environment to support tumor development by regulating cytokines including IL-1β and TNF-α [52]. IL-1β, a proinflammatory cytokine, is associated with tumor progression in lung cancer patients in multiple studies [53]. It has been recently shown that the transcriptional activation of TNF-α, IL-1β, and PTGS2 (COX-2) are among other early oncogenic molecular events that occur in the epithelium of the upper aerodigestive tract under its chronic exposure to known risk factors, such as tobacco smoke N-nitrosamines, with or without nicotine, and/or gastroesophageal refluxate, and can be prevented by the application of specific inhibitors [24,54,55,56,57]. An examination of the Cancer Genome Atlas database shows that for lung cancer, IL-1 and TNF reveal a tendency as an oncogene, as patients with higher IL-1β and TNF-α mRNA levels are linked to shorter overall survival, although this correlation does not reach statistical significance [57]. In another study, it is well documented that TNF-α and IL-1β signaling support COX-2 promoter activity as well as COX-2 and mRNA expressions [58,59]. Moreover, recent studies have demonstrated that COX-2 expression also requires the activity of JNK intracellular signaling [60].

In our study, JNK and COX-2 mRNA in lung tissue and TNF-α and IL-1β protein levels in lung tissue increased in animals as a result of DEN administration. A report on lung tissue and serum demonstrates that DEN treatment increased COX-2, TNF-α, and IL-6 levels [61], while others have shown elevated tumor markers (e.g., carcinoembryonic antigen, CEA) [62]. To the best of our knowledge, the results of the present study were first demonstrating that DEN leads to a high level of JNK expression in lung tissue. With the application of SOR, it was observed that JNK and COX-2 mRNA in lung tissue decrease with the amelioration of tissue damage and inflammatory response. In parallel to this, lung tissue TNF-α and IL-1β levels were decreased with the administration of SOR. It was previously reported that SOR reduces TNF-α and IL-16 levels in liver cancer in vitro [63]. To our knowledge, this is the first attempt to evaluate the effect of SOR on lung JNK and lung TNF-α and IL-1β levels in vivo after DEN administration. However, it has been reported that SOR reduces JNK expression in lung cancer cell lines in vitro [64], and it has therapeutic properties by reducing TNF-α and IL-1β levels in liver cancer [65]. It has also been reported that treatment of low concentrations of SOR significantly inhibits the proliferation of A549 tumor cells in vitro and suppressed tumor growth in vivo [66].

Inflammation also leads to cancer cells dedifferentiating into CSCs through several signaling pathways such as COX-2 and JNK signaling [30,67]. SOX-2 has demonstrated the potential to be a clinically useful biomarker to maintain lung CSCs [33]. There are also studies in the literature reporting that SOX-2 is overexpressed and acts as an oncogene in lung cancer [68,69]. Although the role of SOX-2 in lung cancer has been determined as a result of clinical and preclinical studies [70]. the effect of DEN administration on SOX-2 expression in experimental animals remains unclear. In our study, we observed that DEN increased SOX-2 levels as compared to the control group. In the literature, we could not find any study demonstrating SOX-2 levels in the DEN-induced lung cancer model. However, in line with our result, a recent study declared that the stem cell gene SOX-2 increased significantly in liver tissue with DEN application [71]. In this study, SOR treatment significantly reduced SOX-2 levels in comparison to the DEN group. Consistent with this result, SOR was reported to decrease SOX-2 levels in an in vitro study [72].

## 4. Materials and Methods

### 4.1. Drugs and Reagents

Diethylnitrosamine (DEN) was purchased from Sigma-Aldrich (Cas Number # 55-18-5, St. Louis, MO, USA). Phosphate buffer sodium (PBS) obtained from Sigma-Aldrich (Cas Number # 7558-80-7, St. Louis, MO, USA). Sorafenib (SOR) (BAY-43-9006) was bought by Bayer HealthCare as Nexavar^®^ (İstanbul, Turkey). Ketamine (Cas Number # 1867-66-9) and xylazine (Cas Number # 7361-61-7) were obtained from Sigma. SOX2 (Cat.No.: E-EL-R0882), TNF-α (Cat.No.: E-EL-R2856), and IL-1β (Cat.No.: E-EL-R0012) were obtained from Elabscience (Houston, TX, USA). Hematoxylin Eosin (H&E) was obtained from Merck (Cas Number # 17372-87-1, Darmstadt, Germany). COX-2 (Cat.No.: sc-293182) and JNK (Cat.No.: sc-514539) were obtained from Santa Cruz (Houston, TX, USA). Fluorescein-5-Isothiocyanate (FITC; secondary antibody, Cat.No.: ab6785) and DAPI (Cat.No.: ab104139) were purchased from Abcam (Boston, MA, USA).

### 4.2. Experimental Animals

In total, 30 male albino male Sprague–Dawley rats (250–300 g) were obtained from the Experimental Animal Laboratory of the Medicine and Experimental Application and Research Center of Ataturk University (Erzurum, Turkey). The rats were housed in plastic cages in a well-ventilated room at 24 ± 1 °C, with a normal 12-h light/dark cycle. Commercially available pelleted rat chow and tap water were given ad libitum. The use and care of laboratory animals were accepted by the Atatürk University Institutional Animal Care and Use Committee, and the experiments were performed according to international guidelines (E-42190979-000-2100281403). 

### 4.3. Induction of Lung Carcinogenesis and SOR Treatment Schedule

**Figure 7:**
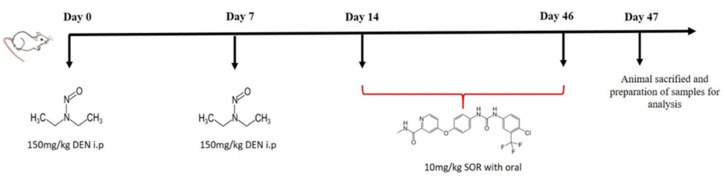
Treatment schedule of the experiment.

Animals were given DEN at a dose of 150 mg/kg body weight intraperitoneally (i.p.) once a week for two weeks. After two weeks, animals were divided into three groups (*n* = 10) and were treated with 10 mg/kg SOR for 42 alternate days orally. The treatment schedule of the experiment was presented in Figure 7.

The dosage of DEN has been determined according to previously published studies [7]. In addition, we supported the optimum dose of DEN with a preliminary assay study with a limited number of animals (4/group). We applied 10 mg/kg SOR daily to rats since higher concentrations were found to be toxic in long-term treatments for rats (pilot experiments and personal communication with Bayer AG) [73,74].

### 4.4. Experimental Design

After the induction of lung cancer, the animals were assigned randomly to three groups (*n*  =  10) as follows:

Group I (Control): Normal control rats

Group II (DEN): DEN-administered rats (150 mg/kg)

Group III (DEN + SOR): DEN-administered rats were treated with 10 mg/kg SOR for 42 alternate days with gavage.

Notably, 24 h after the last treatment, animals were deprived of food overnight and all the rats were anesthetized with 80 mg/kg ketamine + 8 mg/kg xylazine administered i.p. Blood was collected from the jugular vein and serum was separated and used for biochemical investigations. The lung tissues were homogenized in 2 mL of phosphate-buffered saline (PBS; pH 7.4) with TissueLyser II (Qiagen, Hilden, Germany), and the samples were then centrifuged at 14,000× *g* for 15 min at 4 °C. The supernatant solution was separated and stored at −80 °C to measure biochemical parameters. The remaining lung tissues were fixed in a 10% formalin solution for histopathological, immunohistochemical, and immunofluorescence analysis.

### 4.5. Biochemical Analysis

The ELISA plates provided for SOX-2, TNF-α, and IL-1β were pre-coated with an antibody specific to rat SOX-2, TNF-α, and IL-1β, respectively. Firstly, standards and samples were added to the ELISA plates wells and combined with the specific antibody. Then a biotinylated detection antibody specific for rat SOX-2, TNF-α, and IL-1β and Avidin-Horseradish Peroxidase (HRP) conjugate were added to wells and incubated. Free components were removed with washing and substrate solutions were added to each well. Only those wells that contain rat SOX-2, TNF-α, and IL-1β, biotinylated detection antibodies, and Avidin-HRP conjugate appeared blue. The enzyme-substrate reaction was ended by the addition of a stop solution and the color turned yellow. The optical density (OD) of SOX-2, TNF-α, and IL-1β were measured spectrophotometrically at a wavelength of 450 nm. A standard curve was plotted, and an equation was obtained from the absorbance of the standards. The linear SOX-2, TNF-α, and IL-1β concentrations were calculated according to this equation.

### 4.6. Histopathological Analysis

Lung tissues were fixed in 10% buffered formalin for 48 h, dehydrated in a graded ethanol series, and embedded in paraffin. Sections (3–4 µm thick) were placed on slides stained with hematoxylin eosin (H&E) for histopathological analysis and examined under a light microscope (Leica DM 1000, Germany). The sections were evaluated as no (−; 0 cells), mild (+; 1–0 cells), moderate (++; 11–20 cells), and severe (+++; 21 cells and above), according to their histopathological findings [75].

### 4.7. Immunohistochemical and Immunofluorescence Examination

All sections taken on the adherent (poly-L-Lysin) slides were passed through the xylol and alcohol series. The sections were dipped in 3% H_2_O_2_ after washing them in distilled water. Protein blocks were dripped onto the tissues that were boiled with antigen retrieval. For immunohistochemical analysis, tissues were incubated at 37 °C for 30 min with the COX-2 primary antibody. Notably, 3-3′ Diaminobenzidine (DAB) was used as the chromogen. Sections were examined under a light microscope [76].

For the immunofluorescence examination, tissue sections taken on adhesive (poly-L-Lysin) slides were deparaffinized and dehydrated. The sections were kept in 3% H_2_O_2_ for 10 min to inactivate endogenous peroxidase. Then sections were boiled in an antigen retrieval (citrate buffer, pH 6.1) solution to prevent masking of the antigen in the core, and allowed to cool to room temperature for 30 min. After this period, it was incubated for 5 min with protein block compatible with all primer and secondary antibodies to prevent nonspecific ground staining. The JNK primary antibody was kept at the appropriate temperature and time for the conditions of use. FITC was used as a secondary marker. Then, DAPI with DNA marker mounting medium was used. The processed tissues were examined under a fluorescent light microscope (ZEISS AXIO, Oberkochen, Germany).

### 4.8. Statistical Analysis

All statistical analyses were performed using SPSS for Windows 20 (SPSS Inc., Chicago, IL, USA). Shapiro Wilks test was used to assess the normality assumption and Levene Test was used to assess the assumption of homogeneity of variances. Descriptive statistics for continuous variables were expressed as mean ± standard deviation (mean ± SD). The significance of the mean difference between the three groups was investigated by the Welch One Way Analysis of Variance (ANOVA) Test. Games–Howell multiple comparison tests were used for pairwise comparisons. Data were illustrated by the graphical representation. A two-sided *p*-value < 0.05 was considered statistically significant.

For immunohistochemical and immunofluorescence examination, in order to determine the intensity of positive staining from the obtained images, 5 random areas were selected from each image. As a result of the antibody staining used for the evaluation process, the positive/total area was calculated by measuring with the ZEISS Zen Imaging Software program. Data were statistically defined as mean standard deviation (mean ± SD) for % area. To evaluate non-parametric data, the Mann-Whitney U test was performed to compare immunoreactive cells and immunopositive stained areas of positive antibodies with healthy controls. As a result of the test, *p*-value of < 0.05 was considered significant, and the data were presented as mean ± SD.

## 5. Conclusions

**Figure 8:**
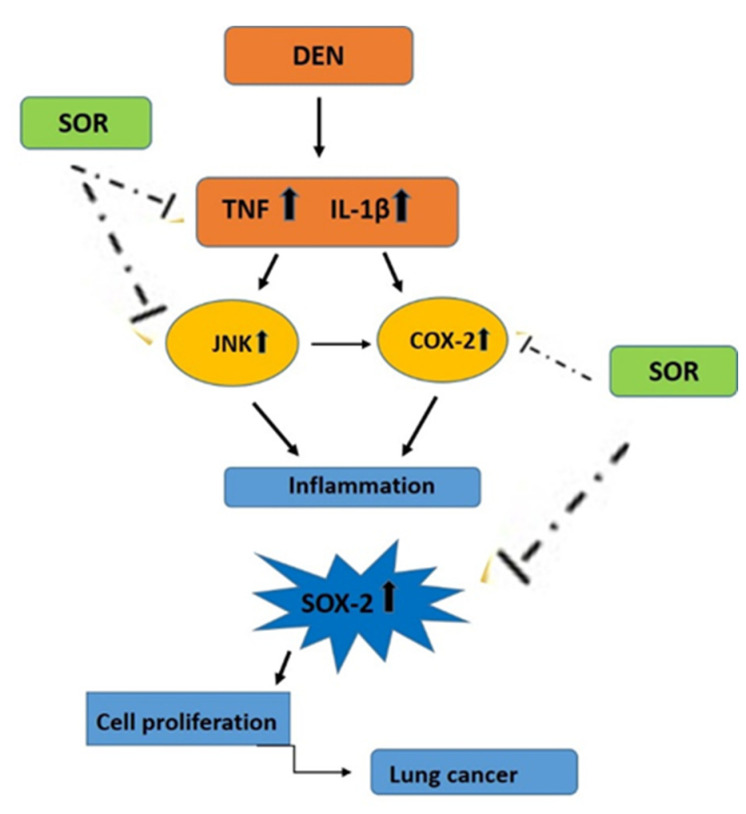
Role of SOR as a potential anticancer agent in lung carcinogenesis via targeting JNK/COX-2 related SOX-2 signaling pathway.

In the present study, we show for the first time that DEN exposure in rats promotes SOX-2 levels through JNK/COX-2 signaling, endowing lung cells with malignant properties Figure 8. These effects of DEN were reversed by the administration of SOR. It was also shown for the first time in this study that SOR reduces inflammation-associated JNK and COX-2 signaling and TNF and IL-1β protein levels in the precancerous microenvironment and decreases the level of SOX-2, which is a marker related to CSCs. These findings are noteworthy because of the close proximity to clinical trials investigating SOR for the treatment of lung cancer. Therefore, we believe that these findings will shed light on studies investigating SOR treatment in lung cancer. However, further studies are required to identify the potential treatment effects of SOR on SOX-2 and its inflammation-related genes in lung cancer progenitor cells will provide further insights into the pathology and new therapeutic avenues for more effective treatment of lung cancer.

## Figures and Tables

**Table 1:** Summary of the histopathological scores based on H&E staining results for lung tissues.

	Control Group	DEN Group	DEN + SOR Group
Peribronchiolar cell infiltrations	−	+++	+
Macrophage in alveolar lumens	−	+++	−
Proliferation and degeneration in the bronchial-bronchiole epithelium	−	+++	+
Hyperemia	−	+++	++

**Table 2:** Scoring immunohistochemical results for lung tissues.

	Control Group	DEN Group	DEN + SOR Group
COX-2	18.26 ± 2.48 ^a^	59.65 ± 1.94 ^b^	32.16 ± 2.71 ^c^

Control: healthy group (no treatment), DEN: diethylnitrosamine group, DEN + SOR: DEN + 10 mg/kg sorafenib. COX-2: Cyclooxygenase-2. a, b, c: the differences between the averages with different letters in the same row are significant. (*p* < 0.05).

**Table 3:** Scoring immunofluorescence results for lung tissues.

	Control Group	DEN Group	DEN + SOR Group
JNK	23.18 ± 3.44 ^a^	63.18 ± 2.67 ^b^	37.86 ± 3.4 ^c^

Control: healthy group (no treatment), DEN: diethylnitrosamine group, DEN + SOR: DEN + 10 mg/kg sorafenib. JNK: c-Jun N-terminal Kinase. ^a^, ^b^, ^c^: the differences between the averages with different letters in the same row are significant. (*p* < 0.05).

## Data Availability

Raw data is available on request.
